# In vitro genotoxicity and cytotoxicity of a particular combination of pemetrexed and cefixime in human peripheral blood lymphocytes

**DOI:** 10.1186/s40064-015-0803-3

**Published:** 2015-01-28

**Authors:** Erman Salih Istifli, Mehmet Topaktaş

**Affiliations:** Department of Biology, Faculty of Science and Letter, Cukurova University, 01330 Adana, Turkey

**Keywords:** Pemetrexed, Cefixime, Genotoxicity, Cytotoxicity, Human peripheral lymphocytes, Synergism

## Abstract

This study aims to find the genotoxic and cytotoxic effects of a particular combination of pemetrexed (PMX) and cefixime (CFX) in human peripheral blood lymphocytes. Chromosome aberration (CA), sister chromatid exchange (SCE), and micronucleus (MN) tests were used to assess genotoxicity. Whereas, the cytotoxicity was evaluated by using mitotic index (MI), proliferation index (PI), and nuclear division index (NDI). Our tests were proceeded with concentrations of 12.5 + 450, 25 + 800, 37.5 + 1150, and 50 + 1500 μg/mL of a mixture of PMX and CFX separately for 24 hr and 48 hr.

The combination of PMX + CFX did not induce the CA or SCE in human peripheral blood lymphocytes when compared with both the control and the solvent control. MN in human peripheral blood lymphocytes was not significantly increased after treatment with a particular combination of PMX + CFX. However, PMX + CFX significantly decreased the MI, PI and NDI at all concentrations for 24- and 48-hr treatment periods when compared with both controls. Generally, PMX + CFX inhibited cell proliferation more than positive control (MMC) and showed a higher cytotoxic effect than MMC at both treatment periods. These results were compared with individual effects of PMX and CFX. As a result, it was observed that a particular combination of PMX + CFX was not genotoxic. However, the combination synergistically increase cytotoxicity in human peripheral blood lymphocytes.

**Electronic supplementary material:**

The online version of this article (doi:10.1186/s40064-015-0803-3) contains supplementary material, which is available to authorized users.

## Introduction

In cytotoxic chemotherapy, patients often receive myelosuppressive doses of antineoplastic agents (Voog et al. 2000). Thus, the majority of patients receiving antineoplastic drugs are potential recipients of antibiotics because of significant myelosuppression that makes them susceptible to bacterial infections. However, it is well-known that drugs regardless of their sequence of administration can interact with each other. The outcome of these interactions can not be predicted based on the individual effect of each drug in their combination. Previous studies on the combination effects of pharmaceuticals with different mechanisms of action have occasionally reported an increasing trend through cytotoxicity. According to Pakulska (1992), benzypenicillin which normally does not demonstrate potential cytotoxic and genotoxic activity (Koseoglu et al. 2004), enhanced the anticancer effect of cyclophosphamide against L1210 leukemia cell line. However, such an interaction was not observed between benzylpenicillin and methotrexate in the same experimental design. Another study by Meurette et al. (2006) demonstrated that TRAIL (TNF-α-related apoptosis-inducing ligand), which failed to induce cytotoxicity in normal human lymphocytes, augmented the cytotoxic activity of 5-fluorouracil and cisplatin in PHA-IL2-activated human peripheral lymphocytes. These authors found that TRAIL-anticancer drug combinations activated a significant cytotoxicity (30-35%) in human peripheral lymphocytes as compared to cytotoxicity elicited by cisplatin (5%) or 5-fluorouracil (10%). A recent study by Jarmalaitė et al. (2008) pointed out that the anti-rheumatic drug infliximab synergistically promoted the cytotoxic activity of methotrexate by decreasing the proliferative ability (measured by proliferation and mitotic index) of peripheral blood lymphocytes from rheumatoid arthritis patients. Thus, we hypothesized that patients receiving antineoplastic drugs could be at risk for potential antineoplastic-antibiotic interactions during the treatment of bacterial infection.

Pemetrexed (PMX) is a folate antagonist that disrupts folate-dependent biosynthetic cycles required for purine and pyrimidine synthesis (Istifli and Topaktas 2013). It is used for the treatment of non-small cell lung cancer, which is the leading cause of cancer related mortality worldwide (Molina et al. 2008). The cytogenetic genotoxicity of PMX is still poorly understood; however, the mechanism is believed to be the misincorporation of uracil base into DNA. Also this aberrant process is associated with increased chromosome breakage (Blount et al. 1997; Weeks et al. 2014). Cefixime (CFX) is a widely prescribed cephalosporin against many gram-negative and gram-positive microorganisms. CFX has a unique chemical formula among other cephalosporins and exerts its bactericidal effect through binding one or more penicillin-binding proteins (PBPs) in the bacterial periplasm (Yotsuji et al. 1988).

To the best of our knowledge, the genotoxic and cytotoxic effects of an antineoplastic-antibiotic combination (PMX + CFX) have not been investigated by an in vitro test system using human peripheral blood lymphocytes so far. Chromosome aberration (CA) test in human peripheral blood lymphocytes is the most widely used cytogenetic marker to detect the effects of DNA-damaging agents (Carrano and Natarajan 1988). Chromatid and chromosome-type CAs can be used to predict the risk of cancer (Hagmar et al. 1994; Hagmar et al. 1998; Hagmar et al. 2004). However, there is evidence that chromosome-type CAs are more robust endpoints to predict cancer as compared to chromatid-type CAs (Bonassi et al. 1995; Bonassi et al. 2000; Boffetta et al. 2007; Liou et al. 1999; Rossner et al. 2005). SCEs are the reciprocal exchanges of DNA between homologous loci of sister chromatids (Gutierrez et al. 1999) and the frequency of SCEs has been used to identify genotoxic agents (Perry and Thomson 1984). There is positive correlation between gene mutations and the increase in SCE (Carrano et al. 1978) and it is well-known that mutagens and carcinogens can induce SCE in different cell types even at concentrations below cytotoxic and carcinogenic limits (Tofilon et al. 1983). MN can be formed as a result of chromosome breaks and dysfunction of mitotic apparatus. Like the CA frequency, epidemiological evidence indicate that in a population consisting of healthy individuals, MN frequency in peripheral blood lymphocytes can be used as a biological marker in the prediction of cancer (Bonassi et al. 2007; Bonassi et al. 2011; Fenech et al. 2011).

The aim of the present study was therefore to investigate the genotoxic and cytotoxic effects of a particular combination of PMX and CFX in human peripheral blood lymphocytes. While SCE, CA, and MN tests were used as the genetic endpoints, the PI, MI, and NDI were calculated to evaluate cytotoxic effect of PMX + CFX.

## Materials and methods

Cukurova University Institutional review board was informed of the protocol to be used with the human subjects, and approved the protocol for the work described prior to the performance of the experiments. In addition, all healthy blood donors gave informed consent for the participation in this study.

### Test samples and chemicals

This study was carried out by using blood samples from four (n = 4 ) healthy volunteer donors (two males and two females, all nonsmokers) aged from 23 to 25 years. Also, the healthy blood donors were not using any medication or dietary supplements throughout the study.

**Figure 1:**
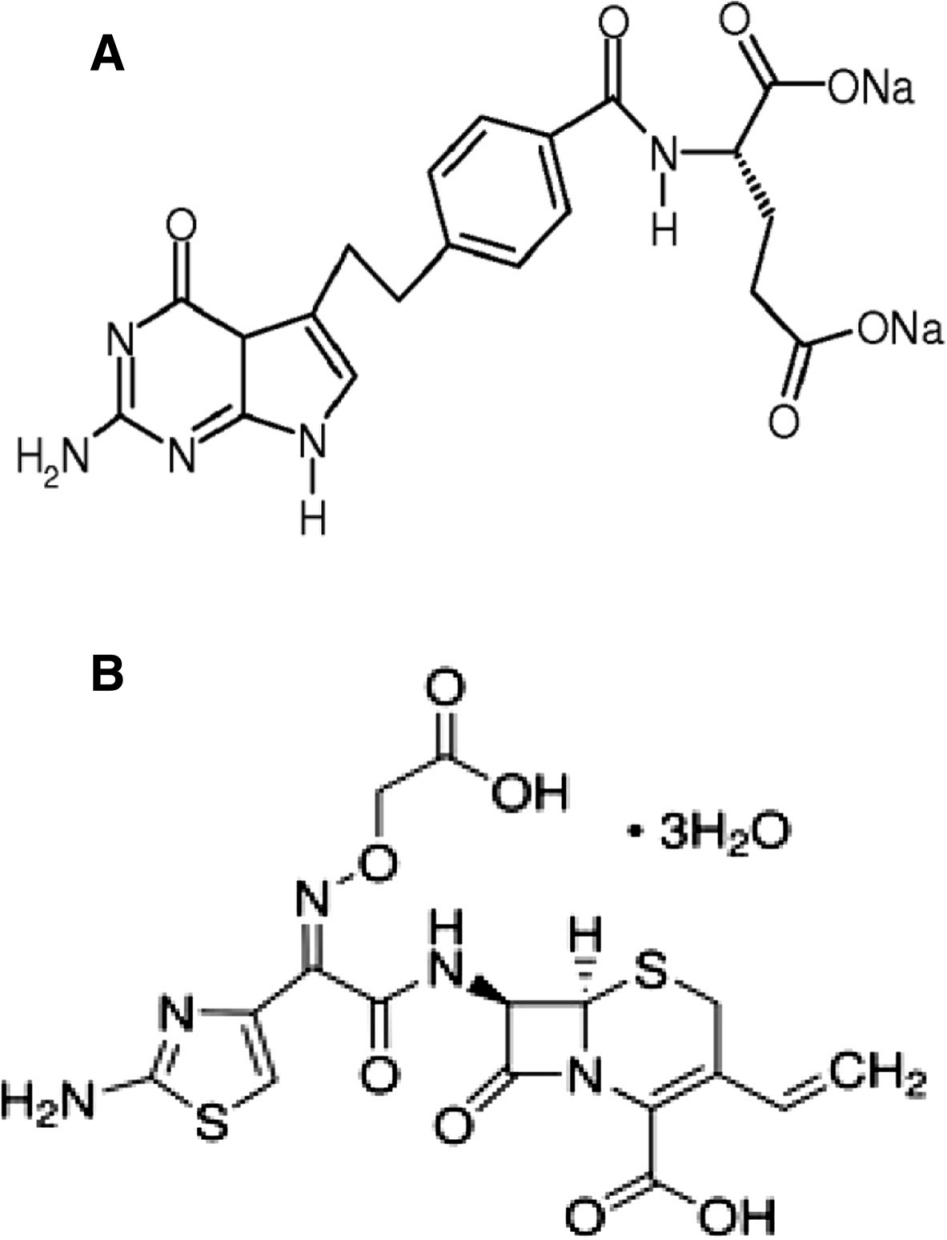
**The chemical structure and formula of Pemetrexed disodium (A) and Cefixime trihydrate (B). (A)** C20H21N5O6 (2R)-2-[[4-[2-(2-amino-4-oxo-1,7- dihydropyrrolo[2,3-d]pyrimidin-5-yl)ethyl]benzoyl]amino]pentanedioic acid (CAS registry number:150399-23-8). **(B)** C16H15N5O7S2 (6*R*,7*R*)-7-{[2-(2-amino-1,3- thiazol-4-yl)-2-(carboxy methoxyimino)acetyl]amino}-3-ethenyl-8-oxo-5-thia- 1- azabicyclo[4.2.0]oct-2-ene-2-carboxylic acid (CAS registry number:79350-37-1).

A commercial formulation of PMX (Pemetrexed disodium [Alimta], containing 500 mg pemetrexed disodium as active ingredient) and CFX (Cefixime, containing 98% cefixime trihydrate as active ingredient) were obtained from local pharmacy and Zentiva (Turkey), respectively. The chemical structures and formulas of PMX and CFX are shown in Figure 1. PMX and CFX were dissolved in sterile bidistilled water and dimethylsulphoxide (DMSO, purity 99%, supplied by Merck - Hohenbrunn, Germany), respectively. Mitomycin-C was used as a positive control (MMC, Kyowa, Hakko, Japan, CAS registry number: 50-07-7) and was dissolved in sterile double-distilled water. 5-Bromodeoxyuridine (B-5002, St. Louis, MO), colchicine (C-9754, St. Louis, MO) and cytochalasin B (C-6762, St. Louis, MO) were purchased from Sigma. Giemsa dye and all other chemicals were purchased from Merck (Darmstadt, Germany). All test solutions were freshly prepared prior to each experiment.

### Concentration selection

In this study, PMX and CFX were tested in combination to evaluate a possible interaction between two drugs. The concentrations of the combination components were chosen according to the individual concentration-finding studies of PMX and CFX. In the previous studies from our laboratory, we investigated the in vitro genotoxic effects of PMX and CFX at concentrations of 25, 50, 75, and 100 μg/mL (Istifli and Topaktas 2013) and 900, 1600, 2300, and 3000 μg/mL, respectively, in human peripheral blood lymphocytes. In the present work, the combinations of half of the single concentrations of PMX and CFX were used as the test concentrations of the drugs. Therefore, the following combinations were tested:

1. 12.5 μg/mL PMX + 450 μg/mL CFX
2. 25 μg/mL PMX + 800 μg/mL CFX
3. 37.5 μg/mL PMX + 1150 μg/mL CFX
4. 50 μg/mL PMX + 1500 μg/mL CFX

The test concentrations of PMX and CFX were prepared on the basis of active ingredient of Alimta (pemetrexed disodium) and cefixime trihydrate, respectively.

### SCE and CA assay

In the present study, human peripheral blood lymphocytes were treated with a combination of PMX and CFX (PMX + CFX). Fresh blood from volunteer donors was collected and transferred to sterile culture tubes containing PB-MAX (GIBCO—Life Technologies, Carlsbad, CA, USA), and was used immediately for the determination of the genotoxicity of PMX + CFX. SCE and CA analyses were conducted using the methods developed by Evans (1984) and Perry and Thomson (1984), with some modifications and this study was organized according to IPCS guidelines (Albertini et al. 2000). Lymphocyte cultures were set up by adding 0.2 mL of whole blood from each of four healthy donors to 2.5 mL of PB-MAX and 5-bromodeoxyuridine (10 μg/mL) was supplemented immediately afterwards. The cultures were incubated at 37°C for 72 h. Serial dilutions of PMX and CFX were made in DMSO (based on the active ingredient) under sterile conditions; thus, DMSO (9 μL/mL) was used as solvent control. A control (untreated control) and a positive control (0.25 μg/mL MMC) were also established for each experiment to ensure the validity of the assay. Treatment times were conducted as 24 h (PMX and CFX were added 48 h after initiating the culture) and 48 h (PMX and CFX were added 24 h after initiating the culture). In order to arrest the cells in metaphase, the cells were exposed to 0.06 μg/mL colchicine 2 h before harvesting. The cells were treated with a hypotonic solution (0.4% KCl) for 15 min at 37°C and then fixed three times in a cold solution consisting of methanol:glacial acetic acid (3:1 v/v) at room temperature. Finally, the centrifuged cells were dropped onto clean slides. The staining of the air-dried slides was performed following the standard methods using 5% Giemsa stain for CA and the modified fluorescence plus Giemsa method for SCE (Speit and Haupter 1985). The slides were irradiated with 30 W, 254 nm UV lamp at 15 cm distance in Sorensen buffer for 30 min, then incubated with 1 × SSC (standard saline citrate) at 60°C for 50 min and stained with 5% Giemsa prepared with Sorensen buffer. One hundred well-spread metaphase per donor were examined to obtain the required number of CAs (a total of 400 metaphase per concentration). Structural and numerical chromosome aberrations within each metaphase were recorded. However, only the structural CAs were taken into consideration to determine the genotoxicity. Percentages of cells with structural chromosomal aberrations were calculated for each donor separately. CAs were classified according to the ISCN (Paz-y-Mino et al. 2002) and evaluated as chromatid-type (breaks and exchanges) and chromosome-type (breaks, fragments, sister chromatid unions, dicentrics, translocations) aberrations. Gaps were not evaluated as CA according to Mace et al. (1978). The scoring of SCE was carried out according to the IPCS guidelines (Albertini et al. 2000). Twenty five well-differentiated second-division metaphase cells were analyzed per donor (a total of 100 second division metaphase for each concentration) for SCE scoring. In addition, a total of 400 cells (100 cells per donor) were scored to determine the PI, which was calculated using the formula: PI = (M1 × 1) + (M2 × 2) + (M3 × 3)/total scored cells. M1, M2, and M3 represent the number of cells undergoing first, second and third mitosis 72-hr cell culture times. In addition, The MI was also determined by scoring 3,000 cells from each donor.

### MN Assay

For the analysis of MN in binucleated lymphocytes, 0.2 mL of fresh blood was used to establish the cultures and the cultures were incubated for 68 hr. Treatment times were conducted as 24 h (PMX and CFX were added 44 h after initiating the culture) and 48 h (PMX and CFX were added 20 h after initiating the culture). Cytochalasin B (Sigma, C6762) was added at 44 hr of the incubation to a final concentration of 6 μg/mL to block cytokinesis. After an additional 24-hr incubation at 37°C, cells were harvested by centrifugation and processed for the MN test in peripheral blood lymphocytes (Rothfuss et al. 2000; Kirsch-Volders et al. 2003). In all subjects, 2,000 binucleated lymphocytes were scored from each donor (8,000 binucleated cells were scored per concentration). A total of 1,000 cells were scored to determine the frequency of the cells with 1, 2, 3, or 4 nuclei and calculate the nuclear division index (NDI) for the cytotoxicity of combination using the formula: NDI = (M1) + (2 × M2) + (3 × M3) + (4 × M4)/N, where M1–M4 represent the number of cells with one to four nuclei and N is the total number of the cells scored (Fenech 2000).

### Statistical analysis

All of the subjects (i.e., the four donors; n = 4), were used as the experimental unit (n) for statistical analysis. Results are expressed as the means ± S.E. (standard error). The multiple comparison of mean data among control, positive control and exposed groups was performed by one-way analysis of variance (ANOVA) and a least significant difference (LSD) was used for inter-group comparisons at p < 0.05. The analysis of interaction between PMX and CFX was performed using CompuSyn 1.0 (ComboSyn, USA), where the combination index (CI) =1 indicates an additive effect, CI < 1 indicates a synergistic effect, and CI > 1 indicates an antagonistic effect according to Chou (2006). In our previous studies on the genotoxicity and cytotoxicity of PMX and CFX, and in this study related to genotoxicity and cytotoxicity of PMX + CFX combination, the same donors were used. All experiments were carried out at the same laboratory and the slides were scored by the same person.

## Results

### Effect of PMX + CFX combination on human lymphocyte culture and its comparison with the negative, solvent and positive control

Four different concentrations and six different parameters (CA, SCE, MN, MI, PI, NDI) were evaluated in two exposure periods (24 and 48 h) to determine the genotoxic and cytotoxic effects of the combination of PMX and CFX on human peripheral blood lymphocytes in vitro.

### Genotoxicity of PMX + CFX combination

The effects of the combination of PMX and CFX on the CAs and MN formation are summarized in Table 1. PMX + CFX did not induce CAs at 24- and 48- hr treatment periods when compared to the negative and the solvent control.

**Table 1:** **Percentage (%) of cells with chromosome aberrations (CAs), %MN, percent of micronucleated binuclear cells (%MNBN) and frequency of SCE in human peripheral blood lymphocytes treated with PMX + CFX for 24- and 48-h treatment periods**

	**Treatment**						
**Test substance**	**Time (h)**	**Concentration (μg/mL)**	**% Cells with CAs ± S.E.**	**MN ± S.E. (%)**	**%MNBN ± S.E.**	**SCE/Cell ± S.E.**	**Min-Max SCE**
Control	-	-	0.50 ± 0.28	0.30 ± 0.09	0.30 ± 0.09	5.56 ± 0.16	1-12
DMSO	24	9 μL	1.75 ± 0.75	0.17 ± 0.07	0.17 ± 0.07	4.54 ± 0.68	1-13
MMC	24	0.25	9.25 ± 1.03	1.72 ± 0.28	1.72 ± 0.28	21.54 ± 1.39	6-47
PMX + CFX	24	12.5 + 450	2.00 ± 0.40 c_3_	0.22 ± 0.02 c_3_	0.22 ± 0.02 c_3_	5.65 ± 0.77 c_3_	1-33
		25 + 800	3.25±0.85 a_1_c_3_	0.12±0.06 c_3_	0.10± 0.04 c_3_	4.28±0.12 c_3_	1-13
		37.5 + 1150	2.50±1.04 c_3_	0.02±0.02 c_3_	0.02±0.02 c_3_	3.77±0.73 c_3_	1-12
		50 + 1500	2.00±0.70 c_3_	0.10±0.04 c_3_	0.10±0.04 c_3_	3.71±0.69 c_3_	1-11
DMSO	48	9 μL	1.00 ± 0.00	0.30 ± 0.14	0.27 ± 0.12	4.11 ± 0.42	1-13
MMC	48	0.25	13.25 ± 1.54	2.90 ± 0.43	2.87 ± 0.44	41.19 ± 4.66	2-78
PMX + CFX	48	12.5 + 450	1.25 ± 0.75 c_3_	0.12 ± 0.02 c_3_	0.12 ± 0.02 c_3_	7.27 ± 1.52 c_3_	1-34
		25 + 800	1.25±0.25 c_3_	0.10±0.04 c_3_	0.10±0.04 c_3_	3.82±0.33 c_3_	1-14
		37.5 + 1150	0.75±0.47 c_3_	0.12±0.04 c_3_	0.12±0.04 c_3_	3.28±0.26 c_3_	1-9
		50 + 1500	0.50 ± 0.50 c_3_	0.05±0.02 c_3_	0.05±0.02 c_3_	4.00±0.17 c_3_	1-8

All data are expressed as mean ± S.E; n = 4.

400 cells were scored per concentration in the CA assay.

4000 cells were scored for the %MNBN.

100 cells were scored per concentration for the SCE assay.

a, significant from control; b, significant from solvent control (DMSO); c, significant from positive control (MMC). a_1_b_1_c_1_: *p* < 0.05; a_2_b_2_c_2_: *p* < 0.01; a_3_b_3_c_3_: *p* < 0.001.

Increasing combination concentrations did not cause a significant increase in the percentage of the binuclear cells with micronuclei (MNBN%) for 24- and 48-hr treatment periods (Table 1). %MN was also not significantly increased when compared with both the negative and the solvent controls in cells treated with PMX + CFX for 24- and 48-hr treatment periods (Table 1).

The observed frequencies of SCE after the addition of PMX and CFX, in peripheral lymphocytes are summarized in Table 1. No significant increase in the mean frequency of SCE values was observed for 24- and 48-hr treatment periods. The positive control MMC significantly induced the SCE in comparison with all concentrations of PMX + CFX (Table 1).

### Cytotoxicity of PMX + CFX combination

In 24- and 48-hr treated cultures MI was found to be significantly reduced when compared with both the negative control and the solvent control. The combination of PMX + CFX decreased the MI to the same extent as the positive control at 12.5 + 450, 25 + 800, 37.5 + 1150 μg/mL, and exerted a greater effect than MMC at 50 + 1500 μg/mL for the 24-h treatment period. Furthermore, PMX + CFX combination showed a greater cytotoxic effect than MMC at all concentrations (12.5 + 450, 25 + 800, 37.5 + 1150, and 50 + 1500 μg/mL) for the 48-h treatment period (Table 2).

**Table 2:** **MI, PI and NDI in human peripheral blood lymphocytes treated with PMX + CFX for 24- and 48-h treatment periods**

	**Treatment**				
**Test substance**	**Time (h)**	**Concentration (μg/mL)**	**MI ± S.E.**	**PI ± S.E.**	**NDI ± S.E.**
Control	-	-	5.11 ± 0.27	2.47 ± 0.03	1.54 ± 0.05
DMSO	24	9 μL	4.39 ± 0.13	2.31 ± 0.04	1.48 ± 0.03
MMC	24	0.25	1.99 ± 0.20	1.74 ± 0.09	1.31 ± 0.03
PMX + CFX	24	12.5 + 450	1.58 ± 0.25 a_3_b_3_	1.91 ± 0.04 a_3_b_2_	1.24 ± 0.03 a_3_b_3_c_1_
		25 + 800	1.70 ± 0.07 a_3_b_3_	1.85 ± 0.09 a_3_b_3_	1.17 ± 0.05 a_3_b_3_c_2_
		37.5 + 1150	1.67 ± 0.34 a_3_b_3_	2.01 ± 0.09 a_3_b_1_	1.17 ± 0.01 a_3_b_3_c_2_
		50 + 1500	1.25 ± 0.20 a_3_b_3_c_1_	1.99 ± 0.14 a_3_b_2_	1.17 ± 0.01 a_3_b_3_c_3_
DMSO	48	9 μL	2.48 ± 0.15	2.33 ± 0.04	1.38 ± 0.04
MMC	48	0.25	1.17 ± 0.19	1.39 ± 0.09	1.22 ± 0.03
PMX + CFX	48	12.5 + 450	0.70 ± 0.18 a_3_b_3_c_2_	1.48 ± 0.07 a_3_b_3_	1.09 ± 0.03 a_3_b_3_c_2_
		25 + 800	0.84 ± 0.17 a_3_b_3_c_1_	1.38 ± 0.05 a_3_b_3_	1.09 ± 0.02 a_3_b_3_c_2_
		37.5 + 1150	0.54 ± 0.19 a_3_b_3_c_2_	1.22 ± 0.05 a_3_b_3_c_1_	1.04 ± 0.01 a_3_b_3_c_3_
		50 + 1500	0.22 ± 0.14 a_3_b_3_c_3_	1.26 ± 0.05 a_3_b_3_c_1_	1.05 ± 0.01 a_3_b_3_c_3_

All data are expressed as mean ± S.E; n = 4.

12000 cells were scored for the MI.

400 cells were scored for the PI.

4000 cells were scored for the NDI.

a, significant from control; b, significant from solvent control (DMSO); c, significant from positive control (MMC). a_1_b_1_c_1_: *p* < 0.05; a_2_b_2_c_2_: *p* < 0.01; a_3_b_3_c_3_: *p* < 0.001.

The combination of PMX + CFX decreased the PI significantly at all concentrations (12.5 + 450, 25 + 800, 37.5 + 1150, and 50 + 1500 μg/mL) in both 24 – and 48-hr treatment periods when compared with the negative control and the solvent control. In addition, the decrease observed in the PI for 48-hr treatment was significantly greater than the positive control MMC at the two highest concentrations (37.5 + 1150 and 50 + 1500 μg/mL) (Table 2).

PMX + CFX combination decreased the NDI significantly for all concentrations and treatment periods when compared with the control groups (Table 2). Furthermore, the combination of PMX + CFX significantly decreased the NDI at all concentrations (12.5 + 450, 25 + 800, 37.5 + 1150, and 50 + 1500 μg/mL) for 24- and 48-h treatment periods when compared with positive control, MMC (Table 2). Thus, PMX + CFX exerted greater inhibitory effect on nuclear division when compared with MMC and showed a higher cytotoxic/cytostatic effect than MMC.

### The comparison of the combination and single treatment effects of PMX and CFX in human peripheral blood lymphocytes

#### Genotoxicity

To compare the combination and single effects of PMX + CFX; the complete results of this study (CAs, SCEs, MN, MI, PI and NDI) and the dataset of the same parameters of single treatments of PMX (Istifli and Topaktas 2013) and CFX are summarized together in Table 3. Generally, in 24-hr treatment, there was no significant difference on the induction of CAs in cultures treated with PMX + CFX as compared to single treatment of CFX; however, CAs were significantly reduced when compared to single treatment of PMX for 24-hr (Table 3). Except two concentrations (12.5 + 450, 50 + 1500 μg/mL), the formation of CAs in 48-hr treatment period was significantly lower than single treatment of PMX, but not CFX. Also, individual exposure of PMX at 25, 50 and 75 μg/mL showed greater percentage of MNBN in 24-hr treatment period than the combination of PMX + CFX at 12.5 + 450, 25 + 800 and 37.5 + 1150 μg/mL. However, in 48-hr treatment, the PMX + CFX combination induced the %MNBN to the same extent with the individual exposure of PMX or CFX. Finally, the PMX + CFX combination (37.5 + 1150 and 50 + 1500 μg/mL) showed reduced frequency of SCE than the individual exposure of CFX but not PMX for 24- and 48-hr treatment periods. Therefore, we conclude that when used in combination, the PMX + CFX combination does not show genotoxic potential.

**Table 3:** **A comparison of the between combination and single effects of PMX and CFX on %cells with CAs, SCE/Cells, %MNBN, MI, PI, and NDI in human peripheral blood lymphocytes for 24- and 48-h treatment periods**

	**Treatment**							
**Test substance**	**Time (h)**	**Concentration (μg/mL)**	**% Cells with CAs ± S.E.**	**SCE/Cell ± S.E.**	**%MNBN ± S.E.**	**MI ± S.E.**	**PI ± S.E.**	**NDI ± S.E.**
PMX^a^	24	25	5.25 ± 0.25	9.52 ± 3.09	0.60 ± 0.07	3.29 ± 0.47	1.79 ± 0.15	1.27 ± 0.05
		50	5.00 ± 1.08	4.73 ± 1.29	0.42 ± 0.14	3.53 ± 0.62	1.75 ± 0.14	1.28 ± 0.06
		75	6.25 ± 0.62	3.67 ± 0.48	0.50 ± 0.12	3.25 ± 0.46	1.96 ± 0.09	1.19 ± 0.02
		100	5.00 ± 1.08	3.42 ± 0.40	0.15 ± 0.06	3.73 ± 0.89	1.72 ± 0.14	1.21 ± 0.04
CFX	24	900	1.25 ± 0.75	5.49 ± 0.39	0.30 ± 0.04	3.20 ± 0.42	2.09 ± 0.09	1.51 ± 0.07
		1600	0.75 ± 0.47	5.37 ± 0.52	0.22 ± 0.06	2.09 ± 0.32	1.84 ± 0.06	1.52 ± 0.04
		2300	0.75 ± 0.25	5.85 ± 0.13	0.25 ± 0.06	2. 31 ± 0.44	1.95 ± 0.08	1.52 ± 0.09
		3000	0.75 ± 0.47	6.09 ± 0.28	0.20 ± 0.07	2.19 ± 0.05	1.86 ± 0.05	1.46 ± 0.04
PMX + CFX	24	12.5 + 450	2.00 ± 0.40 d_2_	5.65 ± 0.77 d_1_	0.22 ± 0.02 d_2_	1.58 ± 0.25 d_1_e_3_	1.91 ± 0.04	1.24 ± 0.03 e_2_
		25 + 800	3.25 ± 0.85 e_1_	4.28 ± 0.12	0.10 ± 0.04 d_2_	1.70 ± 0.07 d_1_	1.85 ± 0.09	1.17 ± 0.05 e_3_
		37.5 + 1150	2.50 ± 1.04 d_2_	3.77 ± 0.73 e_3_	0.02 ± 0.02 d_3_e_2_	1.67 ± 0.34 d_1_	2.01 ± 0.09	1.17 ± 0.01 e_3_
		50 + 1500	2.00 ± 0.70 d_1_	3.71 ± 0.69 e_3_	0.10 ± 0.04	1.25 ± 0.20 d_3_e_1_	1.99 ± 0.14	1.17 ± 0.01 e_2_
PMX^a^	48	25	2.25 ± 1.03	7.14 ± 2.11	0.55 ± 0.11	2.20 ± 0.49	1.88 ± 0.72	1.06 ± 0.21
		50	3.00 ± 0.70	2.87 ± 0.20	0.25 ± 0.06	2.81 ± 0.62	1.65 ± 0.16	1.10 ± 0.26
		75	2.50 ± 0.64	3.32 ± 0.29	0.05 ± 0.03	2.00 ± 0.51	1.70 ± 0.19	1.11 ± 0.02
		100	1.00 ± 0.40	3.05 ± 0.33	0.10 ± 0.04	1.61 ± 0.68	1.53 ± 0.16	1.09 ± 0.24
CFX	48	900	1.25 ± 0.62	7.14 ± 0.90	0.15 ± 0.02	2.18 ± 0.18	1.93 ± 0.14	1.42 ± 0.04
		1600	2.00 ± 0.91	6.23 ± 0.67	0.20 ± 0.04	2.28 ± 0.51	1.96 ± 0.08	1.37 ± 0.06
		2300	1.50 ± 0.64	7.17 ± 1.00	0.20 ± 0.09	2.00 ± 0.52	1.81 ± 0.10	1.35 ± 0.07
		3000	1.75 ± 0.47	7.19 ± 0.99	0.17 ± 0.11	1.70 ± 0.23	1.73 ± 0.08	1.29 ± 0.05
PMX + CFX	48	12.5 + 450	1.25 ± 0.75	7.27 ± 1.52	0.12 ± 0.02 d_3_	0.70 ± 0.18 d_1_e_2_	1.48 ± 0.07 d_1_e_3_	1.09 ± 0.03 e_3_
		25 + 800	1.25 ± 0.25 d_3_	3.82 ± 0.33	0.10 ± 0.04	0.84 ± 0.17 d_2_e_2_	1.38 ± 0.05 e_3_	1.09 ± 0.02 e_3_
		37.5 + 1150	0.75 ± 0.47 d_2_	3.28 ± 0.26 e_2_	0.12 ± 0.04	0.54 ± 0.19 d_1_e_2_	1.22 ± 0.05 d_2_e_3_	1.04 ± 0.01 d_1_e_3_
		50 + 1500	0.50 ± 0.50	4.00 ± 0.17 e_1_	0.05 ± 0.02	0.22 ± 0.14 d_2_e_2_	1.26 ± 0.05 d_3_e_3_	1.05 ± 0.01 e_3_

All data are expressed as mean ± S.E; n = 4.

^a^Istifli and Topaktas 2013.

d, significant from pemetrexed (PMX) separately; e, significant from cefixime (CFX) separately.

d_1_e_1_: *p* < 0.05; d_2_e_2_: *p* < 0.01; d_3_e_3_: *p* < 0.001.

#### Cytotoxicity

On the otherhand, combination exposure of human lymphocytes to various concentrations of PMX and CFX decreased the MI, PI and NDI to the same extent or more than the individual exposure of PMX or CFX (Table 3). Generally, the combination of PMX + CFX decreased the MI and PI significantly at all concentrations in 48-hr treatment period when compared to PMX or CFX alone; however, all the concentrations of the combination decreased the NDI greater than that of the individual exposure of CFX in both treatment times (Table 3).

## Discussion

This is the first study to assess the genotoxicity and cytotoxicity of a particular combination of PMX (commercial formulation) and CFX (active substance) in human peripheral blood lymphocytes.

Our study revealed that the particular combination of PMX and CFX (PMX + CFX) did not increase the frequency of structural CAs or SCEs (Table 1) in any concentration intervals (PMX + CFX; 12.5 + 450, 25 + 800, 37.5 + 1150 and 50 + 1500 μg/mL) and treatment periods (24 and 48 h). Also, when compared with the control groups, the PMX + CFX was not found to significantly induce MN formation as well (Table 1). Even though PMX itself increased the percentage of cells with structural CAs in 24-hr treatment period (Istifli and Topaktas 2013), the PMX + CFX decreased the number of aberrant cells more than the single treatment of PMX (Table 3). That decrease was not a function of an antagonistic interaction between PMX and CFX on the induction of chromosome aberrations due to an enhancement in the cytotoxicity.

The results of this study revealed that the PMX + CFX significantly decreased the MI, PI, and also NDI for all concentrations and exposure periods. This decrease showed a synergistic pattern in 48-hr treatment period (Table 2). The PMX mediated clastogenicity after 24-hr treatment is associated with thymidine nucleotide pool imbalance, DNA topoisomerase II inhibition, and the formation of reactive oxygen species (Tonkinson et al. 1997; Snyder 2009; Buque et al. 2012). DNA damage blocks the entry of cells into S-phase and leads to the activation of DNA repair enzymes. However, the CFX in our pharmaceutical mixture facilitated a faster entry of damaged cells into the S-phase, which refers to the enhancement of the cytotoxicity of PMX. Hence, the reduction of the frequency of cells with CAs was resulted from the death of cells bearing highly damaged chromosomes. In turn, the cells with reduced incidence of chromosome aberrations became dominant. Fairchild et al. (1988) reported that the concurrent addition of hypoxanthine induced normal rates of RNA synthesis and cell cycle progression from G1 to S phase in L1210 cells exposed to MTX for 12 or 24 hr. They concluded that the L1210 cells progressed into cytotoxic S phase instead of being in G1 because of the inhibition of DNA and RNA synthesis by MTX. Our results for the synergistic cytotoxic effect of PMX + CFX are in good agreement with Fairchild et al. (1988).

Generally, the combination with half PMX and CFX concentration, decreased synergistically the MI, PI, and NDI in comparison to individual exposure of each drug. These results are in good agreement with Bareford et al. (2011, 2012) who reported that the antifolate PMX and the multikinase inhibitor sorafenib acted in synergism with the low clinically relevant doses to kill H460, 4 T1, BT474, Huh7, MCF7, and MCF7F cancer cells. They further suggested that PMX and sorafenib killed tumor cells more via a toxic form of autophagy that leads to activation of intrinsic apoptosis pathway. The cephalosporin antibiotic CFX in the current study possesses vinyl and aminothiazole functional groups that are attached to 3′ and 7′ C atoms, respectively. We believe this configuration is related to the enhancement of the cytotoxic effect of PMX. Using zebra fish embryo toxicity testing, Zhang et al. (2013) reported that the toxicity of functional groups attached on the 3′ and 7′ C atoms of cephalosporins (cefaclor, cefaperazone, ceftriaxone, cefepime, ceftizoxime, cefmenoxime and cefmetazole) were positively correlated with the increase in the concentration of the test solution. Although the mechanistic basis of PMX + CFX interaction was not researched in this study, the synergistic cytotoxic effect of PMX and CFX in peripheral blood lymphocytes may depend on the 7-aminothiazole group of CFX (Borzilleri et al. 2006; Das et al. 2006). In the same studies, this 7-aminothiazole group inhibits several cellular protein kinases via a conserved hydrogen-bond interaction. Hence, we think that these structural properties of CFX may contribute to cytotoxicity of PMX by deactivating protein kinases which become activated upon DNA damage prior to the cell cycle arrest to repair the damage.

Synergistic increases in cytotoxicity with the use of COX-2 specific inhibitors, Chk1 inhibitors (PF-00477736), and nitric oxide (NO) (O’Kane et al. 2010; Blasina et al. 2011; Nagai et al. 2012) were also reported in previous studies on the enhancement of PMX cytotoxicity in vitro on various mesothelioma (MSTO-211H, NCl-H2052, NCl-H2452) and human lung adenocarcinoma (A549) cell lines.

## Conclusion

Our results showed that the combination of PMX and CFX exerted synergistic cytotoxic activity, but not genotoxicity, in human peripheral blood lymphocytes. In addition to cellular effects of PMX + CFX, previous studies have indicated a histological level of toxic interaction between methotrexate and penicillin-derivative antibiotics (Williams et al. 1984; Ronchera et al. 1993; Zarychanski et al. 2006). It was confirmed that penicillins could competitively bind to the human organic anion transporter (hOAT) that reduce the tubular secretion of methotrexate in an in vitro mouse model (Williams et al. 1984). Thus, we propose that the interaction of drugs should be rigorously examined to avoid toxicity in clinical practice not only at the cellular but also at the histological level. Taken together, we observed a significant cytotoxic interaction in the mixture of half PMX and half CFX combinations in human peripheral blood lymphocytes. We suggest that the prescription of CFX for bacterial infections in patients receiving PMX could be relatively cytotoxic.

## References

[CR1] Albertini RJ, Anderson D, Douglas GR, Hagmar L, Hemminki K, Merlo F, Natarajan AT, Norppa H, Shuker DE, Tice R, Waters MD, Aitio A (2000). IPCS guidelines for the monitoring of genotoxic effects of carcinogens in humans. international programme on chemical safety. Mutat Res.

[CR2] Bareford MD, Hamed HA, Tang Y, Cruickshanks N, Burow ME, Fisher PB, Moran RG, Nephew KP, Grant S, Dent P (2011). Sorafenib enhances pemetrexed cytotoxicity through an autophagy-dependent mechanism in cancer cells. Autophagy.

[CR3] Bareford MD, Hamed HA, Allegood J, Cruickshanks N, Poklepovic A, Park MA, Ogretmen B, Spiegel S, Grant S, Dent P (2012). Sorafenib and pemetrexed toxicity in cancer cells is mediated via SRC-ERK signaling. Cancer Biol Ther.

[CR4] Blasina A, Hallin JF, Tan W, Gerrit L, Jani JP (2011). Efficacy of the Chk1 inhibitor PF 00477736 and pemetrexed in human mesothelioma.

[CR5] Blount BC, Mack MM, Wehr CM, MacGregor JT, Hiatt RA, Wang G, Wickramasinghe SN, Everson RB, Ames BN (1997). Folate deficiency causes uracil misincorporation into human DNA and chromosome breakage: implications for cancer and neuronal damage. Proc Natl Acad Sci U S A.

[CR6] Boffetta P, van der Hel O, Norppa H, Fabianova E, Fucic A, Gundy S, Lazutka J, Cebulska-Wasilewska A, Puskailerova D, Znaor A, Kelecsenyi Z, Kurtinaitis J, Rachtan J, Forni A, Vermeulen R, Bonassi S (2007). Chromosomal aberrations and cancer risk: results of a cohort study from Central Europe. Am J Epidemiol.

[CR7] Bonassi S, Abbondandolo A, Camurri L, Dal Pra L, De Ferrari M, Degrassi F (1995). Are chromosome aberrations in circulating lymphocytes predictive of future cancer onset in humans? preliminary results of an Italian cohort study. Cancer Genet Cytogenet.

[CR8] Bonassi S, Hagmar L, Stromberg U, Montagud AH, Tinnerberg H, Forni A, Heikkila P, Wanders S, Wilhardt P, Hansteen IL, Knudsen LE, Norppa H (2000). Chromosomal aberrations in lymphocytes predict human cancer independently of exposure to carcinogens. European Study Group on Cytogenetic Biomarkers and Health. Cancer Res.

[CR9] Bonassi S, Znaor A, Ceppi M, Lando C, Chang WP, Holland N, Kirsch-Volders M, Zeiger E, Ban S, Barale R, Bigatti MP, Bolognesi C, Cebulska-Wasilewska A, Fabianova E, Fucic A, Hagmar L, Joksic G, Martelli A, Migliore L, Mirkova E, Scarfi MR, Zijno A, Norppa H, Fenech M (2007). An increased micronucleus frequency in peripheral blood lymphocytes predicts the risk of cancer in humans. Carcinogenesis.

[CR10] Bonassi S, El-Zein R, Bolognesi C, Fenech M (2011). Micronuclei frequency in peripheral blood lymphocytes and cancer risk: evidence from human studies. Mutagenesis.

[CR11] Borzilleri RM, Bhide RS, Barrish JC, D’Arienzo CJ, Derbin GM, Fargnoli J, Hunt JT, Jeyaseelan R, Kamath A, Kukral DW, Marathe P, Mortillo S, Qian L, Tokarski JS, Wautlet BS, Zheng X, Lombardo LJ (2006). Discovery and evaluation of N-cyclopropyl- 2,4-difluoro-5-((2-(pyridin-2-ylamino)thiazol-5- ylmethyl)amino)benzamide (BMS-605541), a selective and orally efficacious inhibitor of vascular endothelial growth factor receptor-2. J Med Chem.

[CR12] Buque A, Muhialdin J, Munoz A, Calvo B, Carrera S, Aresti U, Sancho A, Rubio I, Lopez-Vivanco G (2012). Molecular mechanism implicated in pemetrexed-induced apoptosis in human melanoma cells. Mol Cancer.

[CR13] Carrano AV, Natarajan AT (1988). International commission for protection against environmental mutagens and carcinogens. ICPEMC publication no. 14. considerations for population monitoring using cytogenetic techniques. Mutat Res.

[CR14] Carrano AV, Thompson LH, Lindl PA, Minkler JL (1978). Sister chromatid exchange as an indicator of mutagenesis. Nature.

[CR15] Chou TC (2006). Theoretical basis, experimental design, and computerized simulation of synergism and antagonism in drug combination studies. Pharmacol Rev.

[CR16] Das J, Chen P, Norris D, Padmanabha R, Lin J, Moquin RV, Shen Z, Cook LS, Doweyko AM, Pitt S, Pang S, Shen DR, Fang Q, de Fex HF, McIntyre KW, Shuster DJ, Gillooly KM, Behnia K, Schieven GL, Wityak J, Barrish JC (2006). 2-aminothiazole as a novel kinase inhibitor template. Structure-activity relationship studies toward the discovery of N-(2-chloro-6-methylphenyl)-2-[[6-[4-(2-hydroxyethyl)-1- piperazinyl)]-2-methyl-4-pyrimidinyl]amino)]-1,3-thiazole-5-carboxamide (dasatinib, BMS-354825) as a potent pan-Src kinase inhibitor. J Med Chem.

[CR17] Evans HJ (1984). Human peripheral blood lymphocytes for the analysis of chromosome aberrations in mutagen tests.

[CR18] Fairchild CR, Maybaum J, Straw JA (1988). Enhanced cytotoxicity with methotrexate in conjunction with hypoxanthine in L1210 cells in culture. Cancer Chemother Pharmacol.

[CR19] Fenech M (2000). The in vitro micronucleus technique. Mutat Res.

[CR20] Fenech M, Holland N, Zeiger E, Chang WP, Burgaz S, Thomas P, Bolognesi C, Knasmueller S, Kirsch-Volders M, Bonassi S (2011). The HUMN and HUMNxL international collaboration projects on human micronucleus assays in lymphocytes and buccal cells–past, present and future. Mutagenesis.

[CR21] Gutierrez S, Carbonell E, Galofre P, Creus A, Marcos R (1999). Low sensitivity of the sister chromatid exchange assay to detect the genotoxic effects of radioiodine therapy. Mutagenesis.

[CR22] Hagmar L, Brogger A, Hansteen IL, Heim S, Hogstedt B, Knudsen L (1994). Cancer risk in humans predicted by increased levels of chromosomal aberrations in lymphocytes: Nordic study group on the health risk of chromosome damage. Cancer Res.

[CR23] Hagmar L, Bonassi S, Stromberg U, Brogger A, Knudsen LE, Norppa H, Reuterwall C (1998). Chromosomal aberrations in lymphocytes predict human cancer: a report from the European Study Group on Cytogenetic Biomarkers and Health (ESCH). Cancer Res.

[CR24] Hagmar L, Stromberg U, Bonassi S, Hansteen IL, Knudsen LE, Lindholm C, Norppa H (2004). Impact of types of lymphocyte chromosomal aberrations on human cancer risk: results from Nordic and Italian cohorts. Cancer Res.

[CR25] Istifli ES, Topaktas M (2013). Genotoxicity of pemetrexed in human peripheral blood lymphocytes. Cytotechnology.

[CR26] Jarmalaitė S, Dedonytė V, Mierauskienė J, Šimkutė L, Ranceva J, Butrimienė I (2008). Cytogenetic effects of treatment with methotrexate and infliximab in rheumatoid arthritis patients. Biologija.

[CR27] Kirsch-Volders M, Sofuni T, Aardema M, Albertini S, Eastmond D, Fenech M, Ishidate M, Kirchner S, Lorge E, Morita T, Norppa H, Surralles J, Vanhauwaert A, Wakata A (2003). Report from the in vitro micronucleus assay working group. Mutat Res.

[CR28] Koseoglu V, Kismet E, Soysal Y, Ulucan H, Dundaroz R, Imirzalioglu N, Gokcay E (2004). Investigation of DNA damage in lymphocytes exposed to benzathine penicillin G. Pediatr Int.

[CR29] Liou SH, Lung JC, Chen YH, Yang T, Hsieh LL, Chen CJ, Wu TN (1999). Increased chromosome-type chromosome aberration frequencies as biomarkers of cancer risk in a blackfoot endemic area. Cancer Res.

[CR30] Mace ML, Daskal Y, Wray W (1978). Scanning-electron microscopy of chromosome aberrations. Mutat Res.

[CR31] Meurette O, Fontaine A, Rebillard A, Le Moigne G, Lamy T, Lagadic-Gossmann D, Dimanche-Boitrel MT (2006). Cytotoxicity of TRAIL/anticancer drug combinations in human normal cells. Ann N Y Acad Sci.

[CR32] Molina JR, Yang P, Cassivi SD, Schild SE, Adjei AA (2008). Non-small cell lung cancer: epidemiology, risk factors, treatment, and survivorship. Mayo Clin Proc.

[CR33] Nagai H, Yasuda H, Hatachi Y, Xue D, Sasaki T, Yamaya M, Sakamori Y, Togashi Y, Masago K, Ito I, Kim YH, Mio T, Mishima M (2012). Nitric oxide (NO) enhances pemetrexed cytotoxicity via NOcGMP signaling in lung adenocarcinoma cells in vitro and in vivo. Int J Oncol.

[CR34] O’Kane SL, Eagle GL, Greenman J, Lind MJ, Cawkwell L (2010). COX-2 specific inhibitors enhance the cytotoxic effects of pemetrexed in mesothelioma cell lines. Lung Cancer.

[CR35] Pakulska W (1992). The effect of benzylpenicillin and doxycycline on toxicity and antineoplastic action of cyclophosphamide and methotrexate in mice. Acta Pol Pharm.

[CR36] Paz-y-Mino C, Bustamante G, Sanchez ME, Leone PE (2002). Cytogenetic monitoring in a population occupationally exposed to pesticides in Ecuador. Environ Health Perspect.

[CR37] Perry PE, Thomson EJ (1984). The methodology of sister chromatid exchanges.

[CR38] Ronchera CL, Hernandez T, Peris JE, Torres F, Granero L, Jimenez NV, Pla JM (1993). Pharmacokinetic interaction between high-dose methotrexate and amoxycillin. Ther Drug Monit.

[CR39] Rossner P, Boffetta P, Ceppi M, Bonassi S, Smerhovsky Z, Landa K, Juzova D, Sram RJ (2005). Chromosomal aberrations in lymphocytes of healthy subjects and risk of cancer. Environ Health Perspect.

[CR40] Rothfuss A, Schutz P, Bochum S, Volm T, Eberhardt E, Kreienberg R, Vogel W, Speit G (2000). Induced micronucleus frequencies in peripheral lymphocytes as a screening test for carriers of a BRCA1 mutation in breast cancer families. Cancer Res.

[CR41] Snyder RD (2009). An update on the genotoxicity and carcinogenicity of marketed pharmaceuticals with reference to in silico predictivity. Environ Mol Mutagen.

[CR42] Speit G, Haupter S (1985). On the mechanism of differential Giemsa staining of bromodeoxyuridine-substituted chromosomes. II. differences between the demonstration of sister chromatid differentiation and replication patterns. Hum Genet.

[CR43] Tofilon PJ, Williams ME, Barcellos MH, Deen DF (1983). Comparison of the sister chromatid exchange and cell survival assays as a measure of tumor cell sensitivity in vitro to cis-diamminedichloroplatinum (II). Cancer Res.

[CR44] Tonkinson JL, Marder P, Andis SL, Schultz RM, Gossett LS, Shih C, Mendelsohn LG (1997). Cell cycle effects of antifolate antimetabolites: implications for cytotoxicity and cytostasis. Cancer Chemother Pharmacol.

[CR45] Voog E, Bienvenu J, Warzocha K, Moullet I, Dumontet C, Thieblemont C, Monneret G, Gutowski MC, Coiffier B, Salles G (2000). Factors that predict chemotherapy-induced myelosuppression in lymphoma patients: role of the tumor necrosis factor ligand-receptor system. J Clin Oncol.

[CR46] Weeks LD, Zentner GE, Scacheri PC, Gerson SL (2014). Uracil DNA glycosylase (UNG) loss enhances DNA double strand break formation in human cancer cells exposed to pemetrexed. Cell Death Dis.

[CR47] Williams WM, Chen TS, Huang KC (1984). Effect of penicillin on the renal tubular secretion of methotrexate in the monkey. Cancer Res.

[CR48] Yotsuji A, Mitsuyama J, Hori R, Yasuda T, Saikawa I, Inoue M, Mitsuhashi S (1988). Mechanism of action of cephalosporins and resistance caused by decreased affinity for penicillin-binding proteins in Bacteroides fragilis. Antimicrob Agents Chemother.

[CR49] Zarychanski R, Wlodarczyk K, Ariano R, Bow E (2006). Pharmacokinetic interaction between methotrexate and piperacillin/tazobactam resulting in prolonged toxic concentrations of methotrexate. J Antimicrob Chemother.

[CR50] Zhang J, Qian J, Tong J, Zhang D, Hu C (2013). Toxic effects of cephalosporins with specific functional groups as indicated by zebrafish embryo toxicity testing. Chem Res Toxicol.

